# Supplementary material for: Causal Relationship between Aging and Anorexia Nervosa: A White-Matter-Microstructure-Mediated Mendelian Randomization Analysis
Source: Biomedicines. 2024 Aug 16;12(8):1874. doi: 10.3390/biomedicines12081874 (PMC11351342; doi:10.3390/biomedicines12081874)
Supplement: Supplementary file 1 [file biomedicines-12-01874-s001.zip › Supplementary Figure/Figure S4/Singe SNP plot of MR analysis for TL to ubm-b-2101.pdf]

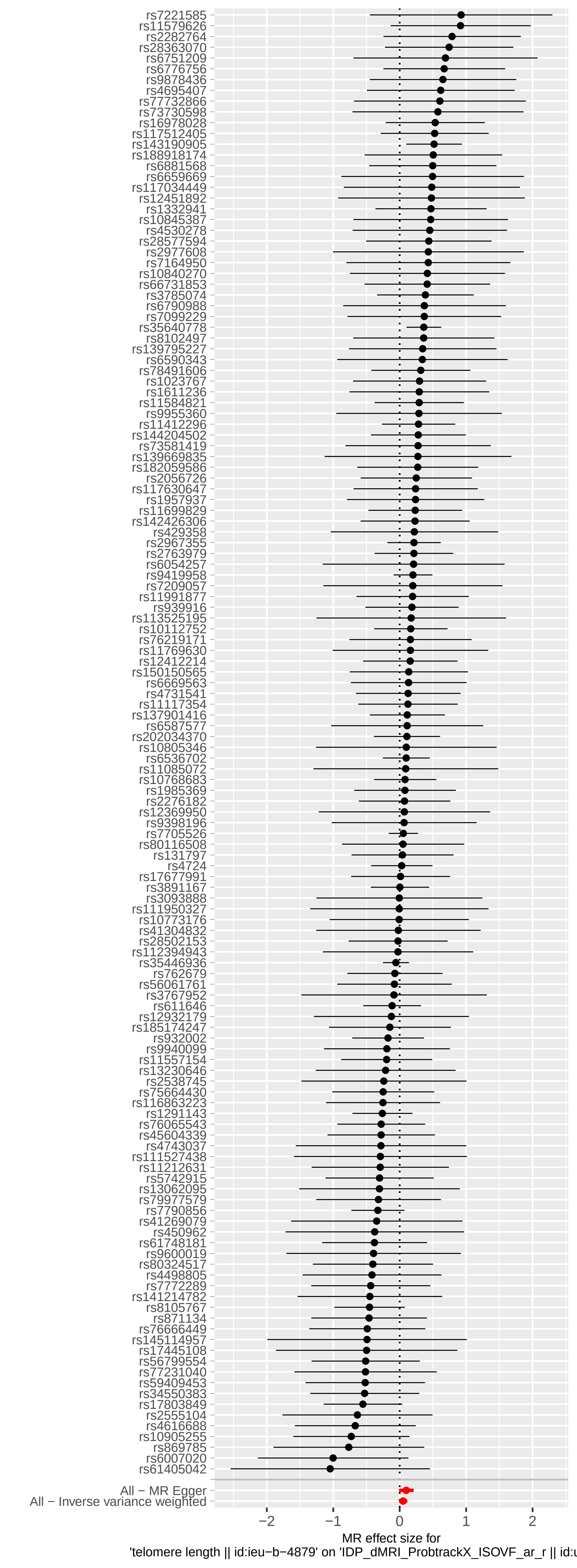

rs7221585
rs11579626
rs2282764
rs28363070
rs6751209
rs6776756
rs9878436
rs4695407
rs77732866
rs73730598
rs16978028
rs117512405
rs143190905
rs188918174
rs6881568
rs6659669
rs117034449
rs12451892
rs1332941
rs10845387
rs4530278
rs28577594
rs2977608
rs7164950
rs10840270
rs66731853
rs3785074
rs6790988
rs7099229
rs35640778
rs8102497
rs139795227
rs6590343
rs78491606
rs1023767
rs1611236
rs11584821
rs9955360
rs11412296
rs144204502
rs73581419
rs139669835
rs182059586
rs2056726
rs117630647
rs1957937
rs11699829
rs142426306
rs429358
rs2967355
rs2763979
rs6054257
rs9419958
rs7209057
rs11991877
rs939916
rs113525195
rs10112752
rs76219171
rs11769630
rs12412214
rs150150565
rs6669563
rs4731541
rs11117354
rs137901416
rs6587577
rs202034370
rs10805346
rs6536702
rs11085072
rs10768683
rs1985369
rs2276182
rs12369950
rs9398196
rs7705526
rs80116508
rs131797
rs4724
rs17677991
rs3891167
rs3093888
rs111950327
rs10773176
rs41304832
rs28502153
rs112394943
rs35446936
rs762679
rs56061761
rs3767952
rs611646
rs12932179
rs185174247
rs932002
rs9940099
rs11557154
rs13230646
rs2538745
rs75664430
rs116863223
rs1291143
rs76065543
rs45604339
rs4743037
rs111527438
rs11212631
rs5742915
rs13062095
rs79977579
rs7790856
rs41269079
rs450962
rs61748181
rs9600019
rs80324517
rs4498805
rs7772289
rs141214782
rs8105767
rs871134
rs76666449
rs145114957
rs17445108
rs56799554
rs77231040
rs59409453
rs34550383
rs17803849
rs2555104
rs4616688
rs10905255
rs869785
rs6007020
rs61405042
All − MR Egger
All − Inverse variance weighted
−2
−1
0
1
2
MR effect size for
'telomere length || id:ieu−b−4879' on 'IDP_dMRI_ProbtrackX_ISOVF_ar_r || id:u
